# Neoadjuvant image-guided helical intensity modulated radiotherapy of extremity sarcomas – a single center experience

**DOI:** 10.1186/s13014-019-1207-2

**Published:** 2019-01-09

**Authors:** Jan C. Peeken, Christoph Knie, Kerstin A. Kessel, Daniel Habermehl, Severin Kampfer, Hendrik Dapper, Michal Devecka, Rüdiger von Eisenhart-Rothe, Katja Specht, Wilko Weichert, Klaus Wörtler, Carolin Knebel, Jan J. Wilkens, Stephanie E. Combs

**Affiliations:** 1 Department of Radiation Oncology, Klinikum rechts der Isar, Technical University of Munich, Ismaninger Straße 22, 81675 Munich, Germany; 2 Department of Radiation Sciences (DRS), Institute of Innovative Radiotherapy (iRT), Helmholtz Zentrum München, Ingolstädter Landstraße 1, Neuherberg, Germany; 3 Department of Orthopaedic Surgery, Klinikum rechts der Isar, Technical University of Munich, Ismaninger Straße 22, 81675 Munich, Germany; 4 Department of Pathology, Klinikum rechts der Isar, Technical University of Munich, Ismaninger Straße 22, 81675 Munich, Germany; 5 Department of Radiology, Klinikum rechts der Isar, Technical University of Munich, Ismaninger Straße 22, 81675 Munich, Germany; 6 Deutsches Konsortium für Translationale Krebsforschung (DKTK), Partner Site Munich, Munich, Germany

**Keywords:** Soft tissue sarcoma, Tomotherapy, IMRT, Extremity, Image-guidance, IGRT

## Abstract

**Background:**

Advanced radiotherapy (RT) techniques allow normal tissue to be spared in patients with extremity soft tissue sarcoma (STS). This work aims to evaluate toxicity and outcome after neoadjuvant image-guided radiotherapy (IGRT) as helical intensity modulated radiotherapy (IMRT) with reduced margins based on MRI-based target definition in patients with STS.

**Methods:**

Between 2010 to 2014, 41 patients with extremity STS were treated with IGRT delivered as helical IMRT on a tomotherapy machine. The tumor site was in the upper extremity in 6 patients (15%) and lower extremity in 35 patients (85%). Reduced margins of 2.5 cm in longitudinal direction and 1.0 cm in axial direction were used to expand the MRI-defined gross tumor volume, including peritumoral edema, to the clinical target volume. An additional margin of 5 mm was added to receive the planning target volume. The full total dose of 50 Gy in 2 Gy fractions was sucessfully applied in 40 patients. Two patients received chemotherapy instead of surgery due to systemic progression. All patients were included into a strict follow-up program and were seen interdisciplinarily by the Departments of Orthopaedic Surgery and Radiation Oncology.

**Results:**

Thirty eight patients that received total RT total dose and subsequent resection were analyzed for outcome. After a median follow-up of 38.5 months cumulative OS, local PFS and systemic PFS at 2 years were determined at 78.2, 85.2 and 54.5%, respectively. Two of 6 local recurrences were proximal marginal misses. Negative resection margins were achieved in 84% of patients. The rate of major wound complications was comparable to previous IMRT studies with 36.8%. RT was overall tolerable with low toxicity rates.

**Conclusions:**

IMRT-IGRT offers neoadjuvant treatment for extremity STS with reduced safety margins and thus low toxicity rates. Wound complication rates were comparable to previously reported frequencies. Two reported marginal misses suggest a word of caution for reduction of longitudinal safety margins.

**Electronic supplementary material:**

The online version of this article (10.1186/s13014-019-1207-2) contains supplementary material, which is available to authorized users.

## Background

Patients with extremity soft tissue sarcomas (STS) are a particular challenge for the interdisciplinary team. Complete resection is crucial for long-term outcome of affected patients. In comparison to amputations, Rosenberg and coworkers reported a comparable overall survival after limb-sparing surgery combined with radiation therapy (RT). As consequence, the combination approach is evaluated carefully in every case and can be considered as standard of care in extremity STS, when feasible [1]. A retrospective SEER analysis showed a survival benefit from RT for high-grade sarcomas, however, it could not be reproduced in prospective trials [2, 3]. Especially in limb tumors, intricate anatomy and the necessity of high local radiation doses bear the risk of treatment-related side effects that can decrease functional outcome and impair quality of life [4, 5].

A matter of discussion is the ideal time point of RT (pre-operative vs. post-operative). The clear advantage of preoperative RT protocols for extremity soft tissue sarcomas is the reduced size of the target volume and lower necessary RT dose. Treatment volumes are significantly smaller compared to the postoperative setting, which leads to reduced morbidity rates in terms of limb edema, joint stiffness, fibrosis and fractures [5]. On the other hand, the risk of acute wound healing complications may be higher when compared to patient groups treated with postoperative RT. A randomized trial under the auspices of the National Cancer Institute of Canada (NCIC) demonstrated that wound complications were twice as high after preoperative radiotherapy compared with postoperative radiotherapy (35% versus 17%) [6].

Improvements in treatment precision and advanced dose applications can optimize the therapeutic window in patients with sarcomas. The development of intensity modulated radiotherapy (IMRT) allows for precise dose modulation to complex target structures. Especially, in combination with image guidance (Image Guided Radiotherapy, IGRT), IMRT leads to improved targeting with the potential to reduce safety margins. Finally, this may result in sparing of normal tissue with potentially lower toxicity rates [7–10]. Helical tomotherapy combines fan-beam IMRT with MV-CT imaging allowing daily image guidance. Several modeling studies in patients with lower extremity sarcomas demonstrated that IGRT/IMRT enables to spare both bone and uninvolved soft tissue [8, 11–14]. Modern imaging capabilities like magnetic resonance imaging (MRI) allow precise treatment planning by taking surrogates for microscopic tumor spread into account. A prospective phase II trial in patients with lower extremity sarcomas evaluated if preoperative MRI-planned IGRT/IMRT can minimize dose to uninvolved tissues, and thus potentially reduce the incidence of acute wound complications. With an incidence rate of 30.5%, major wound complications were less frequent compared to the NCIC trial, though statistically insignificantly [15]. Preoperative IGRT/IMRT significantly diminished the need for tissue transfer. Additionally, chronic adverse effects were lower, but not at a significant level [15]. In a phase II trial conducted by the Radiation Therapy Oncology Group (RTOG) using IGRT to an MRI imaging-based reduced target volume, late side effects were significantly lower compared to the historical cohort of the NCIC trial [16].

In our institution, patients with extremity sarcomas are treated with small-margin IMRT/IGRT in a preoperative neoadjuvant setting within an interdisciplinary sarcoma group. In the present work, we evaluate outcomes in patients treated with helical tomotherapy with focus on wound complications and acute/chronic side effects as well as oncologic outcome.

## Materials and methods

We report on 41 patients with extremity sarcomas treated with neoadjuvant RT using a helical tomotherapy machine after informed consent. Treatment decisions were made in an interdisciplinary setting together with orthopaedic surgeons and radiologists regarding the status of resectability. All patients had high-grade sarcomas yielding an AJCC stage of at least IIA [17].

The patient characteristics are summarized in Table 1. Median age was 58 years (range 21–85 years). In 38 patients (93%), RT was performed at primary diagnosis as neoadjuvant treatment prior to surgery. In 3 patients (7%), RT was performed as neoadjuvant treatment for recurrent sarcomas that did not receive RT initially.

**Table 1:** Patient characteristics

Characteristic	*n*	%
Age (years)		
Mean (range)	58 (21–85)	
≤ 50	10	24
> 50	31	76
Anatomic Site		
Upper extremity	6	15
Lower Extremity	35	85
Deep	41	100
Size		
≤ 5 cm	6	15
5–10 cm	13	32
> 10 cm	9	22
> 15 cm	13	32
Tumor		
Primary tumor	38	93
Recurrent tumor	3	7
Histology		
Undifferentiated Pleomorphic sarcoma	15	37
Myxofibrosarcoma	7	17
Dedifferentiated Liposarcoma	5	12
Leiomyosarcoma	5	12
Myxoid Liposarcoma	4	10
Synovial Sarcoma	3	7
Pleomorphic Rhabdomyosarcoma	1	2
Alveolar soft tissue sarcoma	1	2
Pathologic grade		
2	23	56
3	18	44
Nodal status		
No indication of positive nodes	41	100
Positive	0	0
M-stadium		
M0	39	95
M1	0	0
Mx	2	5

All patients were treated with helical tomotherapy. Tomotherapy was chosen to allow for precise positioning with daily MV-CT-imaging assuring reliable realignment necessary for the reduced treatment margins. Limb fixation was achieved with custom immobilization devices, e.g. using personalized Scotch Cast fixation, or vacuum pillows depending on the location. To allow for target coverage to superficial target regions, in 33 patients, bolus material was wrapped around the target region. Target volume definition was based on CT as well as MR-imaging with and without contrast enhancement. Generally, the gross tumor volume (GTV) was defined as the macroscopic lesion visible on contrast-enhanced T1-weight sequences plus surrounding edematous changes determined from fat saturated T2-weight sequences or short tau inversion recovery (STIR) sequences. To cover regions of microscopic spread, a clinical target volume (CTV) was defined including the GTV extending 2.5 cm proximally and distally of the GTV and 1 cm radially (1.5 cm in one patient due to bone invasion), respecting anatomical barriers. The planning target volume (PTV) was defined by a circumferential safety margin of 0.5 cm around the CTV.

All patients but one were treated with a homogeneous dose to the PTV of 50 Gy in 2 Gy per fraction. In one patient, early salvage resection at 28 Gy total dose was performed due to a local furunculosis and ulcerative tumor progression. In all patients, the treatment concept was planned as neoadjuvant radiotherapy, and surgery was scheduled after a median time interval of 39 days in all 38 patients that received the total dose of neoadjuvant RT (range 31–56 days). Two patients developed distant metastases in between staging and first follow up after RT. Both patients received chemotherapy instead of surgery. All other patients were treated surgically after neoadjuvant RT. In total, 9 patients received chemotherapy (1 before, 6 after RT and surgery, 2 after RT instead of surgery). Regimens included adriamycin and ifosfamide in 6 patients and adriamycin alone in 3 patients.

### Follow-up and statistical analysis

All patients were included in a dedicated follow-up program, and data was collected within the “Wilhem Sander Therapie-Einheit für Weichteilsarkome”. Nineteen patients were enrolled in the ongoing PREMISS trial. The first follow-up after radiotherapy was at 4–6 weeks, followed by three-month intervals based on the surgical intervention and surgery-related follow-up. Patients were seen by the Department of Orthopaedics as well as the Department of Radiation Oncology.

Tumor control was documented with imaging on each follow-up visit. Additional examinations were scheduled as required. Treatment-related side effects were documented according to the Common Terminology Criteria for Adverse Events (CTCAE) Version 4.0. Wound complications were rated following the definition of major wound complications of the NCIC trial [6].

Primary endpoints of this analysis were overall survival (OS), local and distant progression free survival (PFS). OS and PFS were calculated from the last day of irradiation until death or progression. All 38 patients that received full dose radiation and surgery were analyzed for the outcome and wound complications needing surgical revision. Acute and late morbidity were assessed in all 40 patients that received total dose RT as secondary endpoints.

For dosimetric analysis, a volume of interest representing the skin was created as the 4 mm outer rim of the body structure. Absolute dosimetric parameters were determined (see Additional file 1: Table S2).

OS, PFS and major wound complications were determined using the Kaplan-Meier-Method. All analyses were performed using the Graphpad Prism version 5.0c (GraphPad Software Inc., La Jolla, CA, USA). Potential predictive factors for major wound complications were evaluated using logistic regression using R (version 3.4.0) (R core team, Vienna, Austria).

## Results

**Fig. 1:**
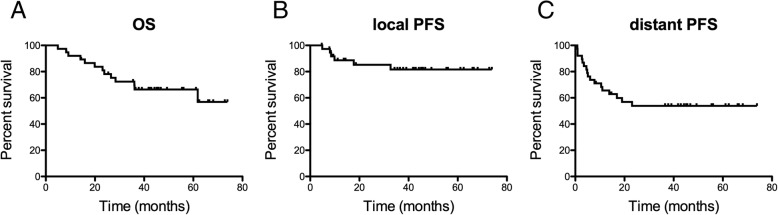
Kaplan Meier survival curves of (**a**) overall survival, (**b**) local progression free survival and (**c**) systemic progression free survival. Data are shown for all 38 patient that received the full neoadjuvant RT regiment (50 Gy total dose with 2 Gy single dose) followed by tumor resection. Label: OS: overall survival, PFS: progression free survival

Forty patients were treated according to the reported regiment. At a median follow up of 38.5 months (range 4–74 months), 13 patients had died (34.2%), 17 patients had developed distant metastases (44.7%), and 6 patients had developed local recurrence (15.7%) of which two were in-field recurrences. The median OS, local and systemic PFS were not reached (see Fig. 1 for Kaplan Meier survival curves). Cumulative OS, local and systemic PFS were 78.2, 85.2 and 54.5% at 2 years and 69.4, 81.6, 53.9, and 53.9% at 3 years, respectively.

All four recurrences were out of field located proximal to the PTV. Two of these recurrences were situated directly adjacent to the field margin (marginal miss). We created two novel PTVs for each patient that had a marginal miss by adding a 4 cm or 5 cm margin in longitudinal direction and a 1.5 cm margin in radial direction to the primary tumor volume defined by the contrast enhancing tumor with an additional 0.5 cm PTV margin. In the first patient, the PTV with a longitudinal margin of 4 cm did not reach the edge of the recurrent tumor, whereas the 5 cm margin PTV touched the edge of the recurrent tumor similarly to our original PTV. In the second patient, both novel PTVs touched the edge of the recurrent tumor similarly to our PTV as the recurrence was situated proximally and radially from the tumor.

A complete resection (R0) was possible in 32 patients (84.2%), R1 resections were performed in 4 patients (10.5%) and RX resections were performed in 2 patients (5.2%). Re-resections were necessary for 7 patients (18.4%) to achieve negative margin status. One patient with R1-resection received high dose rate brachytherapy to the R1-site to a dose of 15 Gy (3 Gy per fraction, twice daily).

Treatment was tolerated well in all patients and was not interrupted due to RT-related toxicities. Moderate side effects of treatment (CTCAE II) included local pain in 11 patients (27.5%), radiation dermatitis within the RT-field in 8 patients (20.0%), fatigue in two patients (5.0%), local edema in two patients (5.0%), alopecia in one patient (2.5%), skin hyperpigmentation in one patient (2.5%) and joint stiffness in one patient (2.5%). The only reported grade III toxicity was radiation dermatitis in one patient (2.5%). There were no grade IV toxicities.

Chronic side effects were assessed during follow-up visits. Moderate chronic side effects (CTCAE II) were reported for fatigue in 5 patients (12.5%), alopecia in 3 patients (7.5%), edema in three patients (7.5%), hyperpigmentation in two patients (5%), pain in one patient (2.5%) and joint stiffness in one patient (2.5%). No bone fractures were reported.

**Fig. 2:**
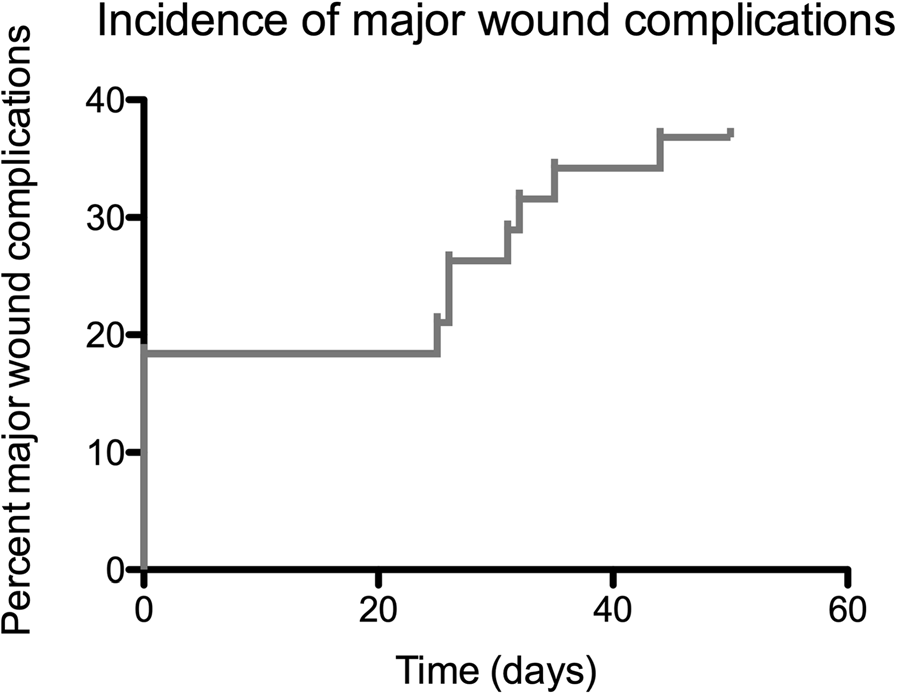
Incidence time curve for the occurence of major wound complications. Data are shown for all 38 patients that received the full neoadjuvant RT regiment (50 Gy total dose with 2 Gy single dose) followed by tumor resection. In 7 patients primary wound closure was not possible requiring secondary wound closure

Major Wound complications, following the definition of the NCIC trial [6], were observed in 13 patients (36.8%) after a median time period of 13 days after surgery (see Additional file 1: Table S1 for individual patient information and Fig. 2 for an incidence curve). Secondary operations for wound repair were necessary in all of these patients with debridement in 9 (69%) patients and operative drainage in one patient (7.6%). In 7 patients primary wound closure was not possible. In total, 9 (69%) patients required secondary wound closure using free flaps or skin grafts. Two patients (16.6%) required deep packing over a period of more than six weeks (performed as vacuum assisted closure) and one patient (7.6%) required an invasive procedure for wound care (without necessity of anesthesia). No patient was readmitted for conservative wound care (e.g. antibiotics). Median time to wound closure after occurrence of major wound complication was 12 days.

In binomial logistic regression for the prediction of major wound complications, “primary wound closure” showed a trend towards significance (odds ratio = − 1.4 (95% confidence interval − 2.9 - 0.011, unadjusted *p*-value = 0.053). No further clinical or dosimetric skin parameter was significantly associated with the occurrence of major wound complication (See Additional file 1: Table S2).

## Discussion

In this retrospective study, we report on a patient cohort with STS that were treated neoadjuvantly with helical IMRT with reduced safety margins after MRI-based target definition using a tomotherapy system. Acute and chronic toxicities were overall low and mostly limited to grade II. Treatment was overall effective with acceptable major wound complications, consistent with previous clinical trials and current literature.

Modern techniques in radiation oncology offer the possibility to create more conformal plans, and reduce dose to normal tissue especially in situations with complex anatomical conditions. Dosimetric studies on IMRT for the treatment of sarcomas have shown reduced dose to relevant structures. Sarcomas of the extremities rarely infiltrate bones, however, segments of the weight bearing bones are exposed to high doses of radiotherapy, which can ultimately lead to treatment-related fractures. Dosimetric comparisons have shown that IMRT has the potential to reduce dose to the bony tissue significantly without compromising target volume coverage. Moreover, dose peaks to soft tissue and skin can be reduced compared to conventional 3D-conformal RT (3DCRT) [8, 11–15]. This was also shown for lower extremity sarcomas, with a substantial increase in conformity index by IMRT [11]. In postoperative RT, likewise, IMRT leads to improved dose conformality compared to 3DCRT [11].

For this effect to translate into clinical efficacy, the combination with image guidance is necessary to ensure precise dose distributions to the correct anatomical positions. Thus, prospective evaluations have addressed the issue of IMRT/IGRT in extremity STS.

Folkert and colleagues compared 3DCRT vs. IMRT for extremity sarcomas and demonstrated a higher local control rate in the IMRT group in spite of a higher number of high-risk feature-patients in that group [18]. Toxicity was comparable between both treatment arms except of radiation dermatitis and edema, which were significantly lower in the IMRT group. Alektiar and colleagues showed similar results for adjuvant and neoadjuvant IMRT. Excellent local control rates were reported with 95% at 2 years, and treatment-related side effects were predominantly grade I and II [19]. Table 2 shows an overview of studies with RT using IMRT with reduced margins in respect to local control and toxicity. It should be noted that the GTV based in this study was defined based on MRI imaging including contrast enhancement and peritumoral edema whereas all studies depicted in defined the GTV as the primary tumor volume based on either CT or MRI.

**Table 2:** Overview of local control and toxicity in prospective trials using intensity modulated radiotherapy (IMRT)

Study	Year	Patients	RT-margins	LPFS	Toxicity (≥ II)
Alektiar et al.	2007	neo: 7 adj: 24	a: 2 cm l: 5 cm	2y 95%	Dermatitis: 26% Edema: 13% Joint stiffness: 19% Neuropathy: 5% Wound Complication: 9.6% Fractures: 6.4%
Alektiar et al.	2008	neo: 7 adj: 34	a: 2 cm l: 3 cm	5y 95%	Edema: 12.2% Joint sitffness: 17.1% Wound complication: 9.9% *chronic*: Dermatitis: 1.9% Fractures: 4.8%
O’Sullivan et al.	2013	neo: 59	a: 1.5 cm l: 4 cm	5y 88%	Wound complications: 30.4% *chronic*: Edema: 11.1% Joint stiffness: 5.6% Fractures: 0%
Folkert et al.	2014	adj: 165	a: 1–1.5 cm l: 3–4 cm	5y 93%	Dermatitis: 31.5% Edema: 8% Joint stiffness: 14.5% *chronic*: Joint stiffness: 12.5% Fracture: 7.9%
Wang et al.	2015	neo: 42^a^	high grade: a:1.5, l: 3 cm low grade: a:1 cm, l: 2 cm	2y 89%	*chronic:* Edema, joint stiffness or fibrosis: 11.9%

label: *a* axial, *adj* adjuvant, *neo* neoadjuvant, *LPFS* Local progression free survival, *l* longitudinal, *RT* radiotherapy, *y* year

^a^partially combined with chemotherapy

Preoperative RT has been shown to have several benefits compared to postoperative adjuvant RT in patients with extremity STS. The main argument against neoadjuvant RT is the elevated rate of surgery-related morbidity in terms of wound complications. This has been shown in several retrospective series, as well as in prospective trials randomizing pre-operative vs. post-operative RT [20–24]. However, the main arguments for pre-operative RT generally outweigh this risk. For example, in the preoperative setting, treated volumes are much smaller, with a lower risk of RT-related side effects [5]. Furthermore, non-manipulated tissue is treated, which assures a higher efficacy of RT resulting in lower total radiation doses [6, 16]. In the preoperative setting, modern IGRT/IMRT irradiation with a tomotherapy system in this study enabled effective treatment with a wound complication rate of 36.8%, which was comparable to a previous prospective IGRT/IMRT study by O’Sullivan et al. [15].

With a 2 year local PFS of 85.2% reported in this study, local control appeared to be slightly lower when compared to similar studies (see Table 2). Even though a low patient number may contribute to this finding, several confounding factors may explain this phenomenon. First, no patient with G1 was included in this cohort in contrast to the RTOG and NCIC trials (G1-rate: 16.5 and 21%, respectively). In addition, the cohort had only deep-seated tumors (RTOG: 81.9%, NCIC: 59%). On the contrary, R1 resection and age were similar to both prospective trials.

In this study, we report four out-of-field recurrences proximal to the PTV including two recurrences directly situated at the PTV margin corresponding to marginal misses. We used reduced expansion margins of 2.5 cm in longitudinal direction and 1.0 cm in radial direction to a GTV including peritumoral edema. This reduced safety margin in longitudinal direction may have missed microscopic spread and may thus have ultimately lead to the observed marginal misses. To further evaluate this, we created two novel PTVs for each patient by adding a 4 cm or 5 cm margin in longitudinal direction and a 1.5 cm margin in radial direction to the primary tumor volume as performed in the prospective trials listed in Table 2. This analysis demonstrated that the local recurrences wouldn’t have been covered by both alternative PTV concepts. In addition, a reduced margin on the basis of a GTV definition including peritumoral edema did not necessarily lead to a reduced absolute margin. The optimal margin reduction for sarcoma irradiation should be assessed in a prospective trial.

The work presented here bears several technical limitations. First of all, the small size of the patient cohort reduces the power of outcome findings, toxicity results and logistic regression analyses. Secondly, the retrospective nature reduces the quality of follow-up information. Thirdly, the majority of patients (80%) were treated using bolus material. Bolus material was not regularly used in the prospective trials discussed above. The use of bolus material aimed to improve superficial dose coverage. On the contrary, increased dose to superficial tissues may increase the risk of wound toxicities. In our study, there was no significant association in logistic regression with major wound complications. However, a more balanced dataset might have enabled a more powerful analysis for the detection of this relationship.

The present work confirms that use of advanced techniques to assure correct daily repositioning in the neoadjuvant setting with smaller safety margins requires elaborate treatment planning but offers low rates of side effects. Tomotherapy offers the possibility of comfortable online imaging with MV-CT as well as conformal dose distributions for complex and extensive volumes in terms of length which are often present in extremity sarcomas [25].

A prospective evaluation of neoadjuvant RT for sarcomas using advanced techniques including daily image guidance to safely reduce safety margins is currently being evaluated within the prospective PREMISS trial in our institution in order to further characterize the value of neoadjuvant RT for extremity sarcomas and to precisely assess toxicity profiles and local recurrences [26].

## Conclusion

Modern RT applications such as helical IMRT offer highly conformal dose distributions even for long and complex target volumes. As a result, toxicity rates and wound complications were overall low. However, two reported proximal marginal misses offer a word of caution for longitudinal margin reduction which should be investigated further in a prospective trial.

## Additional file

Additional file 1: **Table S1.** Individual patients that suffered from major wound complications. **Table S2.** Predictive factors for major wound complications. (DOCX 19 kb)

## Abbreviations

3DCRT: 3D-conformal RT
CTCAE: Common Terminology Criteria for Adverse Events
CTV: Clinical target volume
GTV: Gross tumor volume
IGRT: Image-guided radiotherapy
IMRT: Intensity modulated radiotherapy
MRI: Magnetic resonance imaging
NCIC: National Cancer Center of Canada
OS: Overall survival
PFS: Progression free survival
PTV: Planning target volume
RT: Radiotherapy
RTOG: Radiation Therapy Oncology Group
SIB: Simultaneous integrated boost
STS: Soft tissue sarcoma
