# Supplementary material for: Neoadjuvant image-guided helical intensity modulated radiotherapy of extremity sarcomas – a single center experience
Source: Radiat Oncol. 2019 Jan 9;14:2. doi: 10.1186/s13014-019-1207-2 (PMC6327451; doi:10.1186/s13014-019-1207-2)
Supplement: Supplementary file 1 — Table S1. Individual patients that suffered from major wound complications. Table S2. Predictive factors for major wound complications. (DOCX 19 kb) [file 13014_2019_1207_MOESM1_ESM.docx]

# Additional file 1

## Table S1

## Individual patients that suffered from major wound complications

| **Patient**  **ID** | **Debridement** | **Operative drainage** | **Secondary wound closure^*^** | **Wound management without anesthesia^#^** | **Wound VAC^+^**  **> 6 weeks** |
| --- | --- | --- | --- | --- | --- |
| **1** |  | x |  |  |  |
| **3** |  |  | x |  |  |
| **6** | x | x |  |  | **x** |
| **9** | x |  |  |  |  |
| **13** | x |  | x |  |  |
| **19** | x |  | x |  | **x** |
| **20** |  |  |  | **x** |  |
| **21** | x |  | x |  |  |
| **22** | x |  | x |  |  |
| **27** |  |  | x |  |  |
| **28** | x |  |  |  |  |
| **34** | x |  | x |  |  |
| **36** |  |  | x |  |  |
| **37** | **x** |  | **x** |  |  |

***** Free flaps, skin grafts

# e.g. aspiration

+ vacuum assisted closure

## Table S2 Predictive factors for major wound complications

|  | Odds Ratio  (95% confidence interval) | p-value | adjusted p-value |
| --- | --- | --- | --- |
| Primary wound closure | -1.4 (-2.9-0.011) | 0.053 | 0.74 |
| Wound VAC | 1.3 (-0.078-2.8) | 0.069 | 0.97 |
| Smoking | 1 (-0.65-2.8) | 0.23 | 1 |
| Diabetes | 17 (-470-Inf) | 0.99 | 1 |
| Radiation dermatitis | -3e-16 (-1.3-1.3) | 1 | 1 |
| GTV volume | -0.00092  (-0.0034-0.00086) | 0.38 | 1 |
| Location (upper vs. lower extremity) | 0.65 (-1.2-2.5) | 0.47 | 1 |
| Bolus | 17 (-170-Inf) | 0.99 | 1 |
| Skin Dmax | -0.13 (-0.53-0.2) | 0.47 | 1 |
| Skin V10Gy | 6.6e-06 (-0.0024-0.0023) | 1 | 1 |
| Skin V20Gy | -0.00085 (-0.004-0.0021) | 0.58 | 1 |
| Skin V30Gy | -0.0021 (-0.0068-0.0018) | 0.32 | 1 |
| Skin V40Gy | -0.003 (-0.0096-0.0022) | 0.31 | 1 |
| Skin V50Gy | -0.0033 (-0.017-0.0073) | 0.57 | 1 |

label: Dmax: maximal dose, GTV: gross tumor volume, V: volume, VAC: vacuum assisted closure

The respective factors were tested using binomial logistic regression. P-values were adjusted for multiple testing using the bonferroni method.
